# The Role of Vaginal Mesh Procedures in Pelvic Organ Prolapse Surgery in View of Complication Risk

**DOI:** 10.1155/2013/356960

**Published:** 2013-08-28

**Authors:** David R. Ellington, Holly E. Richter

**Affiliations:** Division of Urogynecology and Pelvic Reconstructive Surgery, Department of Obstetrics and Gynecology, University of Alabama at Birmingham, Birmingham, AL 35233, USA

## Abstract

Synthetic transvaginal mesh has been employed in the treatment of pelvic organ prolapse for more than a decade. As the use of these devices increased during this period so did adverse event reporting. In 2008, the Food and Drug Administration (FDA) Public Health Notification informed physicians and patients of rising concerns with the use of synthetic transvaginal mesh. Shortly thereafter and in parallel to marked increases in adverse event reporting within the Manufacturer and User Device Experience (MAUDE), the FDA released a Safety Communication regarding urogynecologic surgical mesh use. Following this report and in the wake of increased medical industry product withdrawal, growing medicolegal concerns, patient safety, and clinical practice controversy, many gynecologists and pelvic reconstructive surgeons are left with limited long-term data, clinical guidance, and growing uncertainty regarding the role of synthetic transvaginal mesh use in pelvic organ prolapse. This paper reviews the reported complications of synthetic transvaginal mesh with an evidence-based approach as well as providing suggested guidance for the future role of its use amidst the controversy.

## 1. Introduction

The Food and Drug Administration (FDA) cleared the first surgical mesh product specifically designed for the surgical treatment of pelvic organ prolapse (POP) in 2001. Surgical mesh in absorbable and permanent forms had been employed in vaginal approaches to pelvic floor surgery for several years. In fact, biologic grafts and synthetic mesh prostheses have been utilized in abdominal repairs for POP since the 1970s [1]. Nonetheless, with an estimated 300,000 surgical procedures performed annually for prolapse as well as an effort to address high recurrence rates (6 to 29 percent) requiring women to undergo reoperation for POP, efforts to improve patient outcomes has led to the development and introduction of materials, including synthetic mesh, to augment gynecologic reconstructive surgical repairs [2–5]. Despite limited evidence-based data and with the perfect storm of manufacturing company promotion, limitations of the FDA 510(k) process, and with the promise of shorter operative times and minimally invasive techniques, synthetic transvaginal mesh use surged with the development of an estimated 100 synthetic mesh devices.

Unfortunately, concomitant to the increased use of vaginal synthetic mesh was an increase in adverse event reporting, specifically medical device reports (MDRs), within the Manufacturer and User Device Experience (MAUDE) database. In October 2008, the FDA issued a Public Health Notification (PHN) to inform physicians and patients of adverse events related to vaginal reconstructive surgical use of synthetic mesh and to provide recommendations on how to mitigate risks and counsel patients appropriately [1].

Over the next three years, the FDA observed a 5-fold increase in the number of MDRs associated with the use of synthetic vaginal mesh in POP surgery. Thus, in response to these reports and with the impetus to address growing concerns for patient safety, the FDA released a Safety Communication reflecting data from a systematic review of the literature addressing the safety, efficacy, and limitations of existing literature for urogynecologic surgical mesh [6].

Since this time, there has been nothing short of an explosion within the literature regarding the impact the FDA release had on patient, physician, and medical device companies, as well as the concerns for the future role of transvaginal mesh within pelvic reconstructive surgery. Currently, 547,000 web responses are noted using the terms “transvaginal mesh” in the http://www.google.com/ search engine, with an alarming number of those responses referencing litigation. This in combination with challenges of patient counseling, not only for planned pelvic reconstructive surgery utilizing synthetic transvaginal mesh, but for those patients who have previously undergone POP surgery with mesh, adds to the complexity of the issue. Thus, gynecologists and pelvic reconstructive surgeons are currently without robust guidelines for the role of mesh in POP surgery and further limited by a lack of high-quality research addressing long-term patient safety and outcomes.

This report presents a review of the major complications of vaginal mesh use in POP surgery, addresses patient selection considerations, discusses patient counseling issues regarding the role of mesh in POP surgery, and reviews national organizational responses to the FDA Safety Communication as well as recommendations for future direction in POP mesh surgery.

## 2. Complications of Vaginal Mesh Procedures in the Treatment of Pelvic Organ Prolapse

A wide spectrum of potential complications exist with the use of transvaginal mesh in POP surgery. Rare, but severe complications, including death, fistula formation, and mesh erosion into adjacent organs, have been reported in the MAUDE database. Three of seven deaths were related directly to mesh placement procedures and included two bowel perforations and one hemorrhage [7]. Vesicovaginal fistula formation after the use of synthetic transvaginal mesh in the anterior compartment as well as retrovesical hematoma formation and mesh erosions not simply through the vaginal epithelium but into the bladder has also been reported [8, 9]. Though relatively rare, a thorough understanding of these morbid complications and the subsequent management of them is imperative to the consideration of synthetic transvaginal mesh use in POP surgery.

### 2.1. Mesh Exposure

Mesh exposure/erosion and pain are the most commonly reported complications. Brill et al. recently summarized POP adverse events as reported from the MAUDE database (Table 1) [1, 10]. Mesh exposure and mesh extrusion are often utilized interchangeably. IUGA and the International Continence Society have stated that the generic use of the term “erosion” does not necessarily describe all clinical scenarios and complications of synthetic transvaginal mesh implantation and therefore have provided a new terminology and classification system for complications involving transvaginal meshes, tapes, and grafts in the female pelvic floor [6]. Within this classification system, an exposure is defined as vaginal mesh visualized through separated epithelium, whereas a mesh extrusion is the gradual passage of mesh out of the body structure or tissue [11]. Rates of mesh exposure and extrusion complications vary in the literature as does the follow-up rates of the outcome studies from which they are obtained. A recent review of over 1,508 prolapse repair procedures using implanted prostheses revealed a 3% reoperation rate for vaginal mesh erosion in 29 of the 858 procedures performed using transvaginal synthetic mesh. Compared to the posterior and apical compartment, mesh erosions, requiring surgical excision, were more commonly seen after anterior compartment transvaginal mesh repairs [12]. In an update to their original systematic review in 2008, the Society of Gynecologic Surgeons (SGS) analyzed 110 studies reporting adverse events associated with vaginal mesh applications in POP surgery and revealed an overall erosion rate of 10.3 percent [13]. Sixteen of the 110 studies reported on wound granulation tissue development and reported an overall rate of 7.8 percent. Rardin and colleagues recently reported transvaginal mesh erosion rates varying from 0 to 25 percent [14]. Exposure rates for level 1 studies range from 5 to 19 percent [15].

### 2.2. Pain and Dyspareunia

Weber et al. reported a dyspareunia rate of 19 percent following traditional native tissue reconstructive vaginal surgery for prolapse [16]. Dyspareunia rates were reported in 70 of the 110 studies from the SGS review of mesh augmented vaginal repairs with an overall rate of 9.1 percent [13]. Another prospective observational study reported a 20 percent dyspareunia rate after transvaginal synthetic mesh augmentation [17]. As revealed in a recent review of transvaginal synthetic mesh applications for prolapse repair, reported complications of dyspareunia vary from one end of the spectrum to the other [13]. In fact, Nieminen et al. reported a reduction in dyspareunia for patients undergoing transvaginal synthetic mesh anterior wall repairs as compared to traditional anterior repair. Ninety-seven patients were treated with anterior colporrhaphy, and one hundred five were randomized to synthetic transvaginal mesh. The dyspareunia score was lower in the mesh group (*P* = 0.015) [18]. Sentilhes et al. reported no effect of transvaginal synthetic mesh augmentation on sexual function [19]. As noted in IUGA Grafts Roundtable, postoperative vaginal and pelvic pain following either transvaginal mesh or native tissue repairs for POP is difficult to characterized [20]. In a recent review of 23 patients undergoing transvaginal removal of synthetic mesh for mesh-related complications, vaginal pain and dyspareunia were the most noted complications. Excision of synthetic mesh resolved pain in 10 out of the 11 patients [21].

### 2.3. Mesh Contracture

Another unique complication that is often associated with pelvic pain and perhaps a more morbid sequelae is mesh contraction, a shrinkage, or reduction in the size of the vaginal mesh implant that may lead to mesh prominences or strictures within the vagina [22]. A recent case series reported results of seventeen women undergoing surgical intervention for mesh contraction. All of these women presented with severe vaginal pain and focal tenderness over the contracted portions of the mesh. Furthermore, seven of the seventeen women were noted to have vaginal tightness, and five of the seventeen women reported vaginal shortening [6, 17]. Moore and Miklos discuss the importance of avoiding tension on the levator ani muscle and ligamentous attachments as well as minimizing vaginal epithelium excision and maximizing vaginal estrogen both pre- and postoperatively [23]. Ideal graft augmentation and the avoidance of tensioning can still be complicated by scar tissue formation which may vary from patient to patient.

### 2.4. Functional Lower Urinary Tract Sequelae

 The focus of transvaginal mesh implantation is often upon mesh exposures, extrusions, and related or referred pain; however, it is critical to take into account potential risks to the lower urinary tract. In 2007, a Cochrane review of the surgical management of POP included 22 randomized, controlled trials evaluating 2,368 women and noted that approximately 10 percent of women developed new urinary symptoms after surgery for POP. The meta-analysis included traditional repairs as well as abdominal and transvaginal mesh. The impact of POP surgery on lower urinary tract symptoms was limited and inconclusive [24]. Nonetheless, a recent randomized controlled trial comparing anterior colporrhaphy to transvaginal mesh for POP reported a 3.5 percent bladder perforation in the transvaginal mesh arm and 0.5 percent in the colporrhaphy arm. Furthermore, rates of new stress urinary incontinence after surgery were 12.3 percent and 6.3 percent, respectively [25].

## 3. The Future Role of Mesh in Surgical Treatment of Pelvic Organ Prolapse

### 3.1. Patient Selection: Potential Benefit

According to the American Urogynecological Society (AUGS) and American Congress of Obstetrics and Gynecology's (ACOG) recent joint committee opinion, vaginal mesh use in POP should be reserved for high-risk individuals in whom the benefit of mesh placement may justify the risk. These individuals were described as those with recurrent prolapse or medical comorbidities that preclude more invasive and lengthier open and endoscopic procedures [26].

Historically, patients with bothersome symptoms related to POP including, pressure, protrusion, discomfort, and those with associated symptoms or impact on urinary, bowel, or sexual function are considered candidates for intervention [27]. Initial marketing of synthetic vaginal mesh was aimed at improved success rates for POP surgery and time-efficient, minimally invasive procedures. Currently, limited level 1 data exists that clearly guides indications to aid in surgical decision making for the use of synthetic transvaginal mesh augmentation in POP surgery.

Authors from the Consensus of the 2nd International Urogynecological Association's (IUGA) Grafts Roundtable reported that the use of the term “indications” was too strong to employ regarding recommendations reported on the transvaginal mesh use due to the paucity of data in the literature. A summary of potential benefits of synthetic graft use is seen in Table 2 in an adaptation from the terminology used for recommendations from IUGA stratified as “likely to be beneficial,” “possibly beneficial,” “unlikely to be beneficial,” and “not recommended” [20].

Conditions that may be taken into consideration include those in which patients experience repetitive increases in intra-abdominal pressure (chronic bronchitis, chronic constipation, or other frequent Valsalva invoking conditions, such as heavy lifting) [28]. As noted in Table 2, patients with collagen deficiency disorders, denoted as deficient fascia, may also be candidates that would have a likely benefit from transvaginal synthetic mesh repair.

The authors reported that despite the possible circumstances in which mesh use may be appropriate or provide benefit to a patient with POP, they recommend that the patient be fully counseled regarding outcomes and possible complications. Furthermore, as other reviews have demonstrated that there is a timely call for more thorough investigation of surgical mesh use in POP repair, especially studies involving the use of control or native tissue repair arms as well as the inclusion of functional short- and longer-term outcomes with validated symptom and quality of life measures [6].

### 3.2. Patient Selection: Potential Contraindications

There are no definitive evidence-based guidelines regarding absolute contraindications for the use of vaginal mesh in the surgical treatment of POP. Several studies have demonstrated increased risk of mesh exposure and wound infections with increasing BMI; tissue healing may also be impaired in poorly controlled diabetic patients [29, 30]. Thus placement of a vaginal foreign body may not be prudent or indicated. Smoking is associated with decreased vascularity, poor tissue healing, and increased mesh exposure [31]. In one series in which patients underwent abdominal sacral colpoperineorrhaphy, tobacco users were noted to have a 4-fold risk of developing mesh erosions as compared to nonsmokers [32]. Other conditions that should be considered include chronic steroid use and vaginal atrophy [20]. Although not a contraindication, but quite often a concomitant procedure, hysterectomy at the time of anterior vaginal wall mesh augmentation does pose a significant increased risk for mesh exposure [33].

One additional consideration that has been noted in the literature is that of hypersensitivity reactions to synthetic transvaginal mesh. A case-controlled study that included histological and immunohistochemical analysis of vaginal synthetic mesh explanations in a small population of women noted markers of humorally mediated lymphocytic reaction [34]. Though graft-versus-host reactions are not predictable, caution regarding repeat mesh use should be exercised in women who have had previous reactions to transvaginal mesh implantation.

## 4. Patient Counseling/Consenting Process

Patient counseling regarding the use of synthetic transvaginal mesh for the treatment of POP must be individualized. It has been recommended by several national organizations that surgeons placing vaginal mesh have a command of pelvic anatomy as well as undergoing training specific to each device as well as experience with reconstructive surgical techniques [35–37]. Thus, it is equally appropriate, for these same surgeons to conduct patient counseling and consenting. AUGS has provided an excellent resource for physicians (http://www.augs.org/informedconsent/) which equips providers with a transvaginal mesh informed consent toolkit. This resource includes background information regarding the FDA releases, rationale for transvaginal mesh in surgery for POP, and overview of risks and considerations, as well as an informed consent checklist and frequently asked questions. Furthermore, physicians can direct patients to AUGS patient site (http://www.voicesforpfd.org/) to promote education and active dialogue during counseling.

## 5. National Organizational Response to FDA and Future Recommendations

Physicians should continually review the recommendations and publications released by our national organizations regarding transvaginal mesh and POP. Several national gynecological and urological organizations released response statements to provide further guidance amidst the transvaginal mesh controversy following FDA's 2011 release. The National Association For Continence (NAFC) called for the requirement of consistent, specialized training with credentialing by societies for all surgeons performing procedures to treat stress urinary incontinence and/or POP with or without mesh [38]. AUGS responded in support measures as well, recommending thorough patient informed consent as well as an imperative that surgeons performing vaginal mesh procedures undergo training specific to each procedure [37].

The SGS reported general agreement with the recommendations made by the FDA and highlighted that discriminant use of synthetic transvaginal mesh to augment vaginal defects should be performed by trained surgeons with experience in complex reconstructive surgery and only on patients with perceived unacceptable risk of clinical failure using other nonmesh approaches. Furthermore, SGS in parallel with the FDA called for long-term clinical trials evaluating benefits and safety of vaginal mesh placement [36].

ACOG echoed FDA's charge for rigorous comparative effectiveness research as well lending support to the formation of an advisory committee, the Obstetrics and Gynecology Devices Panel. ACOG also recommended voluntary physician reporting through the MAUDE database and noted that complications of vaginal reconstructive surgery also occur with nonmesh approaches [35].

The Society for Female Urology and Urodynamics (SUFU) was also broadly supportive of the FDA white paper and also noted that many of the complications of vaginal mesh surgeries also occur in nonmesh procedures. It was recommended that consideration of mesh placement be conducted on a case by case basis with informed discussion. As with ACOG and AUGS, SUFU also supported a review of the FDA 501k approval process [39].

A summary of the recommendations from AUGS and ACOG's collaborative Committee Opinion, entitled *Vaginal Placement of Synthetic Mesh for Pelvic Organ Prolapse, *released in December 2011 is seen in Table 3 [26].

## 6. Conclusions

Maximizing patients' outcomes and safety while concomitantly minimizing complications is the central concern from which the transvaginal mesh controversy arises. More robust, long-term data and physician-driven event reporting promulgated by the FDA and national gynecological and urological societies will undoubtedly continue to shed light on the future role of transvaginal mesh in pelvic organ prolapse surgery. It is imperative that physicians continue to review the literature, report their own outcomes, and counsel their patients regarding new outcome data, complications, and recommendations from national organizations in women's health regarding prolapse repair. Surgeons with specialized training in native tissue and use of synthetic mesh augmented repairs and management of complications inherent in both groups should lead this charge of delivering care to women affected by POP.

## Figures and Tables

**Table 1:** Reported mesh augmented pelvic organ prolapse surgery adverse events (MAUDE database), 2005–2010.

Rank	Type of event	Medical device reports
1	Erosion	528
2	Pain	472
3	Infection	253
4	Bleeding	124
5	Dyspareunia	108
6	Organ perforation	88
7	Urinary problems	80
8	Vaginal scarring/shrinkage	43
9	Neuromuscular problems	38
10	Recurrent prolapse	32

Brill [10].

**Table 2:** Factors to consider for vaginal mesh use in pelvic organ prolapse surgery.

Variable	Likely benefit	Possible benefit	Unlikely benefit	Not recommended
Age				
<50 years			●	
≥50 years		●		
Recurrent (same site)		●		
Cystocele/anterior compartment				
≥Stage 2		●		
≤Stage 2				●
Posterior compartment			●	
Apex (vault, cuff, and cervix)		●		
Deficient fascia		●		
Chronic increase intra-abdominal pressure		●		
Pain syndromes (local/systemic)				●
Possibility of pregnancy				●
Combination factors				
Recurrent + cystocele > Stage 2	●			
Recurrent + posterior compartment		●		
Recurrent + apex/cuff/cervix	●			
Recurrent + increased abdominal pressure	●			
Recurrent + deficient fascia	●			
Cystocele > Stage 2 + increased intra-abdominal pressure	●			
Cystocele > Stage 2 + Deficient fascia	●			

Adapted from Davila et al. [20].

**Table 3:** Recommendations for the safe and effective use of vaginal mesh for repair of POP.

Outcome reporting for prolapse surgical techniques must clearly define success both objectively and subjectively. Complications and total reoperation rates should be reported as outcomes	
POP vaginal mesh repair should be reserved for high-risk individuals in whom the benefit of mesh placement may justify the risk	
Surgeons should undergo training specific to each device and have experience with reconstructive surgical procedures and a thorough understanding of pelvic anatomy	
Compared to existing mesh products and devices, new products should not be assumed to have equal or improved safety and efficacy unless long-term data are available	
ACOG and AUGS support continued audit and review of outcomes as well as the development of a registry for surveillance for all current and future vaginal mesh implants	
Rigorous comparative effectiveness randomized trials of synthetic mesh and native tissue repair and long-term followup are ideal	
Patients should provide their informed consent after reviewing the risks and benefits of the procedure as well as discussing alternative repairs	

Adapted from ACOG/AUGS [26].
